# Marine Diatom *Skeletonema costatum* Dietary Supplementation Improves Growth, Immunological Responses, Antioxidant Activities, Gene Expressions, and Digestive Enzymes of Shrimp *Litopenaeus vannamei*

**DOI:** 10.1155/anu/5013608

**Published:** 2025-07-09

**Authors:** Mohamed Ashour, Einar Ringø, Mohamed M. Mabrouk, Ahmed I. A. Mansour, Mohammed A. E. Naiel, Abdallah Tageldein Mansour, Rabee M. A. Gheetas, Ehab Mohamed, Ahmed F. Abdelhamid

**Affiliations:** ^1^ Aquaculture Division, National Institute of Oceanography and Fisheries (NIOF), Cairo, 11516, Egypt, azhar.edu.eg; ^2^ Faculty of Biosciences, Fisheries and Economics, Norwegian College of Fishery Science, UiT The Arctic University of Norway, Tromsø, Norway, uit.no; ^3^ Fish Production Department, Faculty of Agriculture, Al-Azhar University, Cairo, 11823, Egypt, azhar.edu.eg; ^4^ Department of Animal Production, Faculty of Agriculture, Zagazig University, Zagazig, Egypt, zu.edu.eg; ^5^ Animal and Fish Production Department, College of Agricultural and Food Sciences, King Faisal University, Al-Ahsa, Saudi Arabia, kfu.edu.sa; ^6^ Animal Production, Faculty of Agriculture, Al-Azhar University, Cairo, 11823, Egypt, azhar.edu.eg; ^7^ Department of Integrative Agriculture, College of Agriculture and Veterinary Medicine, United Arab Emirates University, Al Ain, P.O. Box 15551, Abu Dhabi, UAE, uaeu.ac.ae

**Keywords:** antioxidant activities, feed additives, growth performance, pacific whiteleg shrimp, *Skeletonema costatum*

## Abstract

This work highlighted the impact of dry concentrations (0, 1, 2, and 4 g/kg diet) of the marine diatom species *Skeletonema costatum* (SK_0%_, SK_0.1%_, SK_0.2%_, and SK_0.4%_, respectively) as an aquafeed additive on the postlarvae of Pacific whiteleg shrimp (*Litopenaeus vannamei*). During an 8‐week feeding trial, the impact of *S. costatum* on growth, digestive enzymes, antioxidant activities, immunity‐associated gene expressions, and immunological responses of Pacific white shrimp was investigated. The protein, lipid, and carbohydrates of *S. costatum* were 33.50%, 35.95%, and 11.87%, respectively. Shrimp reared in group SK_0.4%_ exhibited a significant improvement in growth performance indicators, compared to the other groups. The results showed that, compared to the control group (SK_0%_), shrimp‐fed *S. costatum* supplemented groups (SK_0.1%_, SK_0.2%_, and SK_0.4%_) showed significantly (*p*  < 0.05) higher amylase activities. Compared to the control group (SK_0%_), shrimp‐fed *S. costatum* groups (SK_0.1%_, SK_0.2%_, and SK_0.4%_) demonstrated major significant (*p* < 0.05) improvement in catalase and superoxide dismutase values. Shrimp reared in group SK_0.4%_ showed a significant (*p* < 0.05) improvement in lipase and lysozyme activities. The increasing levels of *S. costatum* dietary supplementation significantly (*p* < 0.05) increase the relative gene expression of the p53‐like protein isoform delta (*p53*) gene. SK_0.4%_ revealed a major significant (*p* < 0.05) increase in the relative gene expression of the prophenoloxidase (*PPO1*) and peroxiredoxin (*Prx*) genes. This study highlights the potential of *S. costatum* as a promising aquafeed additive enhancing growth performance and immunity of Pacific whiteleg shrimp health, opening the way for more sustainable shrimp aquaculture practices.

## 1. Introduction

The global aquaculture industry has significantly grown in recent decades as a result of the increasing global demand for seafood products, particularly Pacific whiteleg shrimp (*Litopenaeus vannamei*) [1]. Among the various marine species cultivated, whiteleg shrimp *L. vannamei* comes as the most economically important and widely farmed shrimp species [2]. Its rapid growth rate, high feed conversion efficiency, and adaptability to a wide range of environmental conditions make Pacific whiteleg shrimp an ideal candidate for marine aquaculture production [3].

However, despite its commercial importance, the sustainability and profitability of Pacific whiteleg shrimp farming are often threatened by factors including low quality of water quality, low survivability, reduced growth performance, and suboptimal immune responses, which can all negatively impact the Pacific whiteleg shrimp health, growth, and overall productivity [4, 5]. In response to these challenges, researchers and aquaculturists have increasingly focused on improving Pacific whiteleg shrimp aquafeed additives to enhance their growth performance, immune responses, high survivability, and overall productivity [6].

Using natural feed additives in shrimp diets is one viable strategy because they are known to enhance shrimp health while reducing the environmental effect of aquaculture operations. Microalgae are one of the many natural feed additives that have shown great promise as a nutritional supplement to improve the performance and well‐being of cultured shrimp. Microalgae have prominent fatty acids, vitamins, amino acids, and other bioactive substances that might improve aquatic animals’ immune response, feed utilization, and growth performance [7].

Diatom, particularly marine diatom, is rich in bioactive materials such as polysaccharides, proteins, vitamins, and minerals, which are beneficial for aquatic organisms [8]. *Skeletonema costatum*, a filamentous marine diatom species, has been identified as a significant diatom species with high nutritional value and various potential health benefits for aquatic organisms [9]. The unique chemical and biochemical composition of *S. costatum*, including its high level of antioxidants, polysaccharides, and pigments [10], makes it an attractive supplement for enhancing shrimp performance, especially in marine hatcheries. Recent studies have highlighted the potential of *S. costatum* to develop metamorphosis, improve growth, enhance feed utilization, improve immune responses, and improve resistance to environmental stress in several marine species, such as Pacific whiteleg shrimp [11] and giant tiger prawn (*Penaeus monodon*) [12, 13].

Until now, the application of *S. costatum* as a dietary supplement for Pacific whiteleg shrimp is still relatively underexplored, and there is a need for research to assess its impact on various aspects of shrimp nutrition. While *S. costatum* is extensively utilized in shrimp hatcheries as live feed for shrimp larvae and crustaceans, there is no comprehensive work investigating the impact of *S. costatum* as a feed additive in Pacific whiteleg shrimp cultivation. This study examines how a supplement of *S. costatum* to Pacific whiteleg shrimp diet affects its growth, nutritional consumption, carcass structure, digestive enzymes efficiency, antioxidant levels, immunological response, and gene expression.

## 2. Materials and Methods

### 2.1. Marine Diatom, *S. costatum*

#### 2.1.1. Isolation and Culture Conditions

*S. costatum* is a diatom strain that was previously isolated from the Mediterranean Coast (31°130 4800 N; 29°530 1200 E), at the location of NIOF, Alexandria Branch of Egypt. After isolation, the pure strain was identified [14–16]. *S. costatum* has cell sizes ranging from 2 to 7 µm [17]. The isolate was cultivated indoors at the Microalgae Room, Invertebrate Aquaculture Lab. at the Alexandria Branch of NIOF. Using F/2 standard Guillard media [18], *S. costatum* was cultivated in a batch culture system using conical flasks (2 L) under standard growth parameters, including temperature, salinity, and regular illumination (24 ± 2°C, 34 ± 1 ppt, and 3500 ± 500 Lux/24 h, respectively), under constant aeration. To obtain the *S. costatum* biomass, at a late exponential phase (LEP), after 5 days of starting culture, the cells of microalgae were then harvested via centrifugation (3000 × *g*, 10 min), and then, collected, dried (45°C/48 h), and subsequently kept at −20°C for future utilization.

#### 2.1.2. Biochemical Composition and Phytochemical Content

At the LEP, 10 mL of culture was centrifuged (3000 × *g*, 10 min) to determine the biochemical analysis of protein, carbohydrate, and lipid. The quantification of total protein and total carbohydrates was analyzed as described by Rausch [19] and Myklestad and Haug [20], respectively. The determination of total protein and total carbohydrates was conducted as described by Vautherin and Brink [21] and DuBois et al. [22], respectively. Total lipid was determined by the method described by Bligh and Dyer [23]. The phytochemical content profile of *S. costatum* was determined by gas chromatography–mass spectrometry analysis (GC–Mass) as described by Ashour et al. [24]. Peak identifications were based on comparing the acquired mass spectra to those available in the NIST Library.

### 2.2. Pacific Whiteleg Shrimp

#### 2.2.1. Experimental Techniques

Pacific whiteleg shrimp postlarvae (PL) (0.052 ± 0.001 g and 0.907 ± 0.010 cm) were obtained from a commercial hatchery and transferred to Baltim Research Station, Kafr El‐Sheikh, NIOF. For 15 days, the PL were adapted to the experimental conditions and fed a commercial shrimp basal diet, the control diet (four times daily). 600 PL were allocated into four groups. Each group consisted of 50 PL, three replicates per group. The PL were set in net hapas (70 × 70 × 100 cm) secured in a concrete pond (400 × 200 × 100 cm). The hapa was cleaned daily, and 10% of the total water culture was exchanged daily. Water quality parameters, salinity, dissolved oxygen, and temperature (27 ± 1 ppt, 5.5 ± 0.5 mg/L, and 27.5 ± 1.5°C, respectively) were daily measured following the guidelines of APHA [25]. Moreover, ammonia (NH_3_), nitrite (NO_2_), and nitrate (NO_3_) were weekly determined, and the values were in the recommended ranges of shrimp aquaculture (0.09 ± 0.02, 0.08 ± 0.07, and 0.15 ± 0.06 mg/L, respectively), according to [26].

#### 2.2.2. Experimental Diet and Procedures

Throughout the feeding experiment (8‐weeks), the PL were divided into four dietary groups. Group one (SK_0%_) received a standard commercial shrimp diet as a control (Aller‐Aqua, Giza Governorate, Egypt) and contains protein, carbohydrate, lipid, fiber, and ash at levels of 45%, 34%, 7.9%, 3.65%, and 9.1%, respectively. The remaining three groups (SK_0.1%_, SK_0.2%_, and SK_0.4%_) were enriched with 1, 2, and 4 g of dried marine diatom *S. costatum*, respectively. The procedure for incorporating *S. costatum* into each diet group was carried out as described by [1]. In brief, the standard shrimp diet was ground finely and divided into four equal parts. Subsequently, each part was supplemented with its appropriate quantity of *S. costatum* and carefully blended until a uniform mixture was achieved. After homogenization, a pellet mill was used to pelletize each food group to the appropriate size. After that, these pellets were dried in an oven set to 40°C until their moisture content was below 10%. Subsequently, the pellets were stored (4°C) until required for feeding trial purposes.

#### 2.2.3. Growth Performance Achievement

The weights of PL were measured at the initial (IW, g) and final (FW, g) to calculate shrimp’s growth and nutrient utilization using the following formulas:

Weight gain (WG, g)=Final body weight g−Initial body weight g,

Specific growth rate SGR% /day=ln final body weight−ln initial body weightt×100,

Feed conversion ratio FCR=Total consumed feedWGSurvival rate SR,%=The total final survived number of shrimpThe initial number of shrimp×100,

Feed efficiency ratio FER=WG gFeed intake g.

#### 2.2.4. Samples Collection

After 24‐h fasting period, after the experiment ended, 18 samples were randomly selected, six samples per replicate. The shrimp were killed by overdose of clove oil (450 mg/L) [27] and used for several subsequent analysis, proximate body composition, digestive enzyme activities (amylase and lipase), antioxidant activity indices, and nonspecific immunity. In summary, tissue organs were dissected, weighed, and homogenized in a phosphate buffer solution (pH 7.4) after randomly chosen samples were killed. After centrifuging the tissues for 20 min at 3.500 rpm, the supernatants were carefully obtained.

#### 2.2.5. Shrimp’s Carcass Analysis

Six shrimp samples were randomly selected by the lottery method from each replicate for total protein, ether extract, dry matter, and ash analysis. Before undergoing additional analysis, the samples were mixed, dried, powdered, and stored (−20°C). The chemical constituent of the carcass was examined following AOAC [28] guidelines.

#### 2.2.6. Digestive Enzyme Activities

Following the manufacturer’s instructions, the colorimetric assay was applied to measure the digestive enzymes’ activity. Biodiagnostic Company, Egypt, manufactured specific amylase and lipase kits. The wavelengths at which lipase (580 nm) and amylase (660 nm) were measured according to [29] and [30], respectively.

#### 2.2.7. Antioxidant Activities

Following the manufacturer’s instructions, the colorimetric method was applied to assess the antioxidant activities. Catalase (CAT), malondialdehyde (MDA), and superoxide dismutase (SOD) activities have been investigated. MDA, CAT, and SOD‐specific kits were purchased from the Biodiagnostic Company in Egypt. CAT, MDA, and SOD have amusement wavelengths of 510 [31], 534 [32], and 560 nm [33], respectively.

#### 2.2.8. Lysozyme Activities

Based on the manufacturer’s instructions (SunLong Biotech Co., Ltd., China), the colorimetric method was used to assess the lysozyme (LZM) activity using ELISA kits. During the experiment, the lysozyme‐containing sample was incubated with *Micrococcus lysodeikticus* cells. The reaction was monitored following the protocol by Harshbarger et al. [34] by measuring the drop in absorbance at 450 nm to estimate the rate of substrate conversion.

#### 2.2.9. Immunity‐Related Genes Expressions

Three whole shrimp samples (five individuals per treatment) were preserved in RNA later reagent (Sigma–Aldrich; 1:5 v/v) at −20°C, following the procedure by Goncalves et al. [35]. RNA extraction was conducted using the TRIzol reagent (TRIzol; Life Technologies). The RT2 First Strand Kit was utilized during the extraction to remove DNA. Following, the primer genes used in this study as well as the housekeeping gene are presented in Table 1. Gene expression was conducted utilizing the 2^−ΔΔCt^ protocol, as described by Livak and Schmittgen [39].

**Table 1:** Primers sequences for the shrimp’s qPCR study.

Gene	Nucleotide primers	Accession No.	Amplification length (bp)	References
*P* *r* *x*	$\begin{array}{c} F:CATCTTCAAGGGCACTGCTG\\ R:CGGCCTTCATTGTCTTGGAG \end{array}$	*GQ*995702	139	Liu et al. [36]
*P* *P* *O* *I*	$\begin{array}{c} F:CCAGCAGCGTCTTCTTTACC\\ R:GTTCAATTTCTCGCCCAGGA \end{array}$	*AY*723296	122	Wang et al. [37]
*p*53	$\begin{array}{c} F:CCAAGCAGCAATGTGTCAGT\\ R:TCAGGCTGCCACTTCTTGAT \end{array}$	*KX*827274	190	Nuñez‐Hernandez et al. [38]
*L*5*H*	$\begin{array}{c} F:ATGCTCATCGTTGAAACCCG\\ R:TCGTGTTTTGAAATGACCTTGG \end{array}$	*KF*193065	124	Wang et al. [37]
*β* − *a* *c* *t* *i* *n*	F: ACTGGGACGACATGGAGAAG *R* : *C* *A* *G* *G* *A* *A* *T* *G* *A* *G* *G* *G* *C* *T* *G* *G* *A* *A* *C* *A*	*JF*288784	121	Wang et al. [37]

*Note*: p53, p53‐like protein isoform delta; β‐actin, A housekeeping gene.

Abbreviations: L5H, hemocyanin subunit L5; PPO1, prophenoloxidase; Prx, peroxiredoxin.

### 2.3. Statistical Analysis

Before the statistical assessment started, fundamental presumptions of normality and uniformity were conducted using Levene’s test, and the findings (%) were arc‐sin transformed [40]. Data were calculated in three replications (mean ± standard division). The statistical assessment was carried out by SPSS Statistics Software, conducting a one‐way ANOVA, and Duncan [41] method at a significance level of *p* ≤ 0.05. The figures were generated by GraphPad (Prism 8) Statistics Software [42]. The polynomial regression was applied by Excel software.

## 3. Results

### 3.1. Marine Diatom, *S. costatum*

#### 3.1.1. Biochemical Composition

The biochemical compositions of protein, lipid, and carbohydrates of *S. costatum* were 33.50%, 35.95%, and 11.87%, respectively, at LEP (Table 2).

**Table 2:** Biochemical composition analysis (%) of marine diatom *S. costatum*.

Biochemical analysis	Values (% of dry weight)
Total protein	33.50 ± 1.12
Total lipid	35.95 ± 0.70
Total carbohydrates	11.87 ± 1.15

#### 3.1.2. Phytochemical Compounds Content

The GC–MS findings of *S. costatum* showed nine retention times, based on the peak areas (Table 3 and Figure 1). Table 3 shows nine phytochemical compounds belonging to six phytochemical groups. Retention times of less than 1% were neglected. The highest major group was fatty acids (three retention time (RT) with 84.84% of total peak area (PA)) and consisted of three fatty acids: myristic acid (tetradecanoic acid, 29.68%), palmitoleic acid (cis‐9‐hexadecenoic acid, 16.01%), and palmitic acid (n‐hexadecanoic acid, 39.15%). The second major group was fatty acid derivatives (two RT with 5.38% of total PA): tetradecanoic acid, 12‐methyl‐, methyl ester (fatty acid methyl ester, FAME, 3.59%), and 9‐hexadecenoic acid, 9‐octadecenyl ester, (Z, Z)‐ (fatty acid occtadecenyl ester, FAOE, 1.79%). The third major group was saturated tetracyclic steroid (one RT with 3.74% of total PA): cholestan‐3‐ol, 2‐methylene‐, (3*β*, 5 *α*). The fourth group was triacylglycerol and trilinolein (one RT with 2.75% of total PA). The fifth group was macrocyclic lactones (one RT with 1.43% of total PA) and milbemycin b, 13‐chloro‐5‐demethoxy‐28‐deoxy‐6,28‐epoxy‐5‐ (hydroxyimino)‐25‐ (1‐methylethyl)‐, (6R, 13R, 25R). Finally, the sixth group was carotene (one RT with 1.39% of total PA) and zeaxanthin.

Figure 1GC mass spectra of bioactive compounds identified in diatom isolate *S. costatum*. (A) Zeaxanthin, (B) tetradecanoic acid (myristic acid), (C) cholestan‐3‐ol, 2‐methylene‐, (3*β*, 5*α*)‐, (D) 9‐Hexadecenoic acid, 9‐octadecenyl ester, (Z, Z)‐, (E) trilinolein, (F) milbemycin b, 13‐chloro‐5‐demethoxy‐28‐deoxy‐6,28‐epoxy‐5‐ (hydroxyimino)‐25‐ (1‐methylethyl)‐, (6R, 13R, 25R), (G) tetradecanoic acid, 12‐methyl‐, methyl ester, (H): cis‐9‐hexadecenoic acid (palmitoleic acid), and (I) n‐hexadecanoic acid (palmitic acid).

**Figure:**
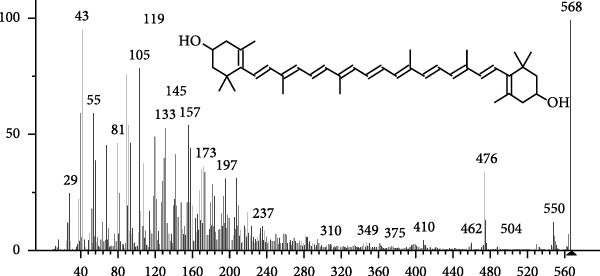
(A)

**Figure:**
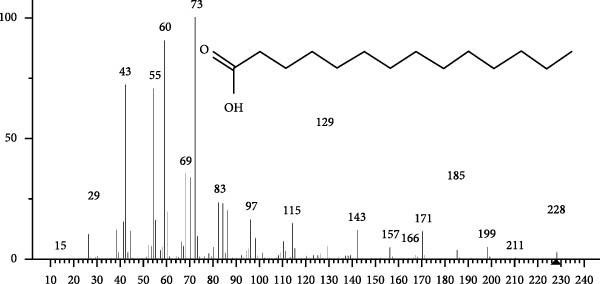
(B)

**Figure:**
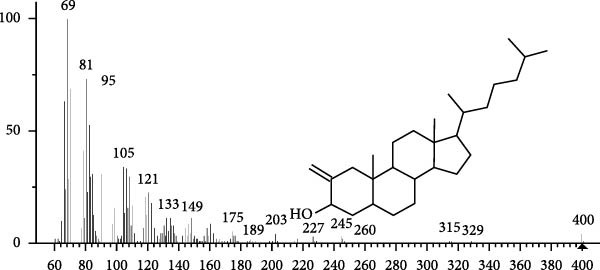
(C)

**Figure:**
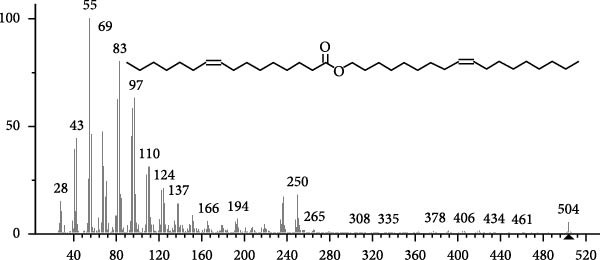
(D)

**Figure:**
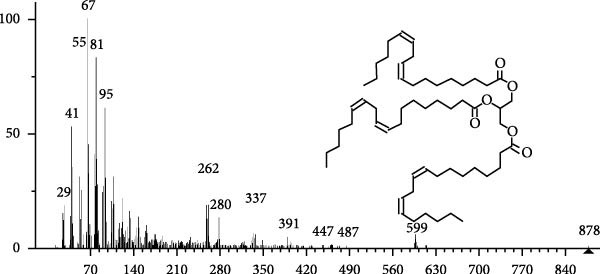
(E)

**Figure:**
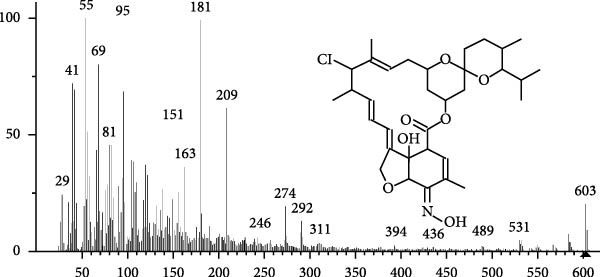
(F)

**Figure:**
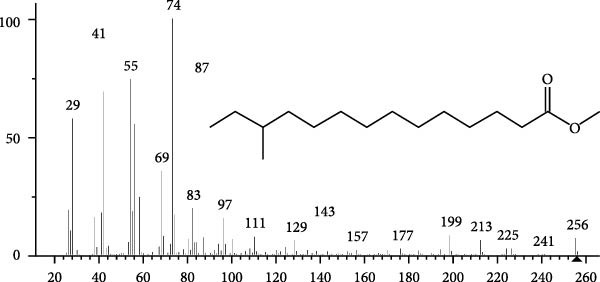
(G)

**Figure:**
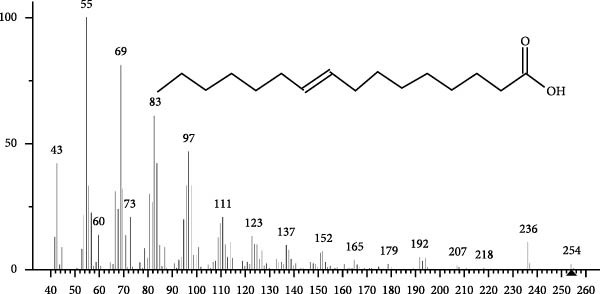
(H)

**Figure:**
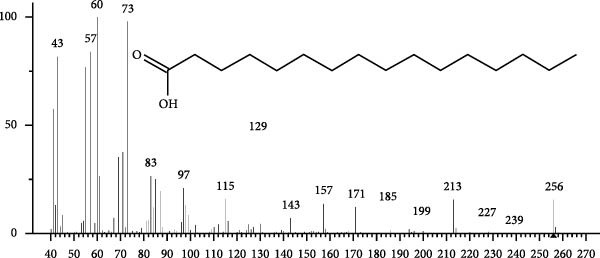
(I)

**Table 3:** Phytochemical reported in *S. costatum*.

RT	PA (%)	Compound name	CF	MW	Nature	Biological activities	Refs
17.35	1.39	Zeaxanthin	C_40_H_56_O_2_	568	Carotene	Antioxidant, anticancer	[43, 44]
18.13	29.68	Tetradecanoic acid (myristic acid)	C_14_H_28_O_2_	228	FA	Antioxidant, Antifungal	[45]
19.3	3.74	Cholestan‐3‐ol, 2‐methylene‐, (3*β*, 5*α*)‐	C_28_H_48_O	400	Saturated tetracyclic steroid	Anticancer, antiviral (SARS‐CoV‐2)	[46, 47]
19.83	1.79	9‐Hexadecenoic acid, 9‐octadecenyl ester, (Z, Z)‐	C_34_H_64_O_2_	504	FAOE	Antimicrobial	[48]
20.07	2.75	Trilinolein	C_57_H_98_O_6_	878	Triacylglycerol	Antioxidant	[49–51]
20.52	1.43	Milbemycin b, 13‐chloro‐5‐demethoxy‐28‐deoxy‐6,28‐epoxy‐5‐ (hydroxyimino)‐25‐ (1‐methylethyl)‐, (6R, 13R,25R)	C_33_H_46_ClNO_7_	603	Macrocyclic lactones	Aquatic animals immunity enhancer	[52]
20.85	3.59	Tetradecanoic acid, 12‐methyl‐, methyl ester	C_16_H_32_O_2_	256	FAME	Antioxidant, antimicrobial, anti‐inflammatory	[53]
21.37	16.01	cis‐9‐Hexadecenoic acid (Palmitoleic acid)	C_16_H_30_O_2_	254	FA	Antioxidant, antiviral, anti‐inflammatory	[54, 55]
21.74	39.15	n‐Hexadecanoic acid (Palmitic acid)	C_16_H_32_O_2_	256	FA	Antioxidant, anticancer, anti‐inflammatory	[56]

*Note:* Retention times those less than 1% were neglected.

Abbreviations: CF, chemical formula; FA, fatty acid; FAEE, fatty acid ethyl ester; FAME, fatty acid methyl ester; FAOE, fatty acid octadecenyl ester; MW, molecular weight; PA, peak area (%); RT, retention time.

### 3.2. Pacific Whiteleg Shrimp

#### 3.2.1. Growth Indicators

Table 4 demonstrates the growth and nutrient utilization indicators of Pacific whiteleg shrimp fed the experimental diets. The SK_0.4%_ group showed a significant improvement (*p*  < 0.05) in FW, WG, survival growth rate (SGR), feed conversion ratio (FCR), feed efficiency ratio (FER), and survival rate (SR), compared to the other groups (SK_0%_, SK_0.1%_, and SK_0.2%_). This improvement was achieved with the increased *S. costatum* supplementation levels. Moreover, significant achievements were found in FL and LG in groups SK_0.4%_ and SK_0.2%_, compared to SK_0%_ (control group) and SK_0.1%_.

**Table 4:** Growth and nutrient indicators of Pacific whiteleg shrimp‐fed diets supplemented with *S. costatum*.

Indices	Groups			
	SK_0%_	SK_0.1%_	SK_0.2%_	SK_0.4%_
IW	0.052 ± 0.001	0.052 ± 0.001	0.052 ± 0.001	0.052 ± 0.001
IL	0.907 ± 0.010	0.897 ± 0.012	0.897 ± 0.012	0.907 ± 0.015
FW	3.897 ± 0.050^b^	4.020 ± 0.082^b^	4.097 ± 0.055^b^	4.413 ± 0.208^a^
FL	8.400 ± 0.100^c^	8.700 ± 0.200^b^	9.133 ± 0.058^a^	9.300 ± 0.100^a^
WG	3.847 ± 0.050^b^	3.970 ± 0.082^b^	4.047 ± 0.055^b^	4.363 ± 0.208^a^
LG	7.480 ± 0.105^c^	7.803 ± 0.190^b^	8.237 ± 0.064^a^	8.393 ± 0.115^a^
SGR	3.337 ± 0.021^b^	3.370 ± 0.001^b^	3.387 ± 0.006^b^	3.443 ± 0.049^a^
FCR	2.237 ± 0.055^a^	2.143 ± 0.031^ab^	2.073 ± 0.049^b^	1.957 ± 0.097^c^
FER	0.447 ± 0.011^c^	0.467 ± 0.007^b^	0.483 ± 0.011^ab^	0.512 ± 0.027^a^
SR	84.66 ± 8.02^b^	88.45 ± 3.16^ab^	87.28 ± 5.03^ab^	91.66 ± 2.06^a^

*Note:* SK_0%_, SK_0.1%_, SK_0.2%_, and SK_0.4%_: diets supplemented with 0, 1, 2, and 4 g of marine diatom *S. costatum*/kg diet, respectively. The provided data were means ± SD (*n* = 3). Significant differences exist between (*p* < 0.05) letters in the same row. The lack of letters indicates no significant.

**Figure 2:**
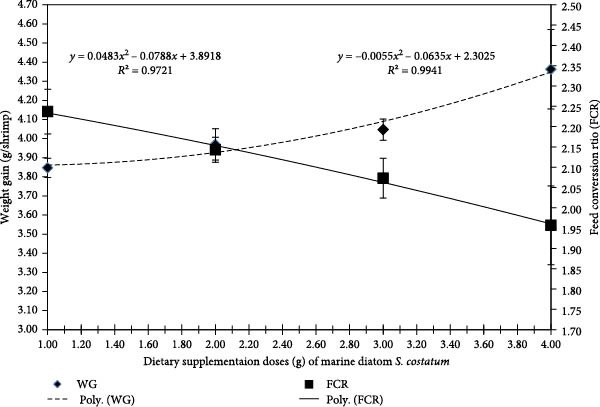
Dietary supplementation levels of marine diatom *S. costatum*.

Figure 2 displays the polynomial regression between FCR and WG and concludes that with the increase of *S. costatum* dietary supplementation level, the polynomial regression of WG was increased (*r* ^2^ = 0.9721) while the polynomial regression of FCR was decreased (*r* ^2^ = 0.9941).

#### 3.2.2. Carcass Analysis

Figure 3A–D reveals the biochemical carcass composition of Pacific whiteleg shrimp‐fed dietary supplemented with several levels (SK_0%_, SK_0.1%_, SK_0.2%_, and SK_0.4%_) of *S. costatum*. As Figure 3A shows, no significant (*p* < 0.05) improvement in the ash content of Pacific whiteleg shrimp between all experimented groups (SK_0%_, SK_0.1%_, SK_0.2%_, and SK_0.4%_). On the other hand, a significant (*p* < 0.05) decrease was reported in dry matter content with the increase of *S. costatum* supplementation levels, as presented in Figure 3B. The percentages of total protein and ether extract significantly (*p* < 0.05) increased with the increase of *S. costatum* supplementation levels (Figure 3C,D).

Figure 3Biochemical carcass composition of shrimp‐fed dietary supported with *S. costatum*. SK_0%_, SK_0.1%_, SK_0.2%_, and SK_0.4%_: diets supplemented with 0, 1, 2, and 4 g of marine diatom *S. costatum*/kg diet, respectively. The provided data were means ± SD (*n* = 3). Significant differences exist between (*p* < 0.05) letters in the same row. The lack of letters indicates no significance. (A) Ash (%), (B) dry matter (%), (C) total protein (%), and (D) ether extract (%).

**Figure:**
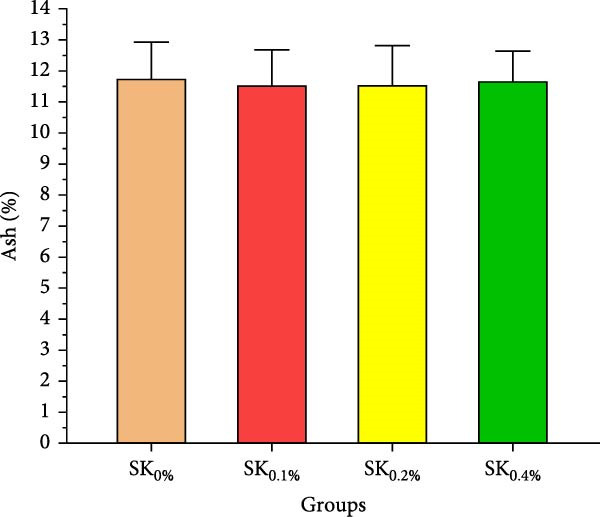
(A)

**Figure:**
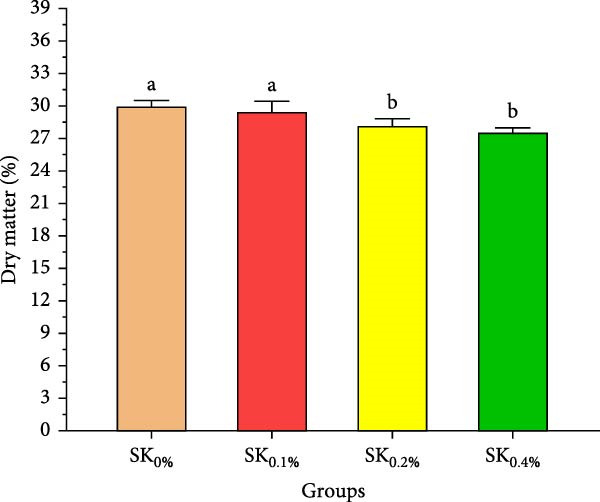
(B)

**Figure:**
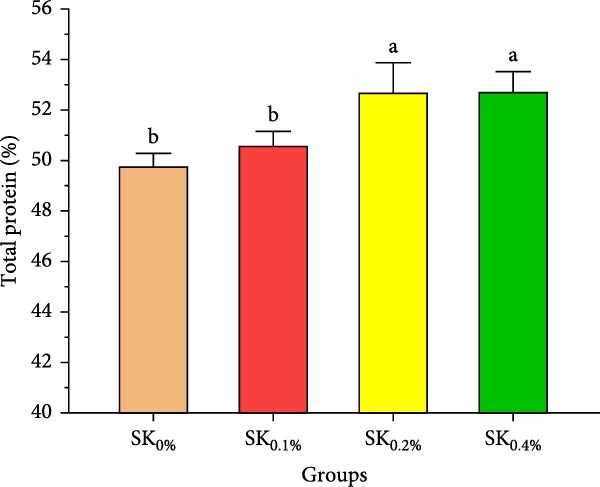
(C)

**Figure:**
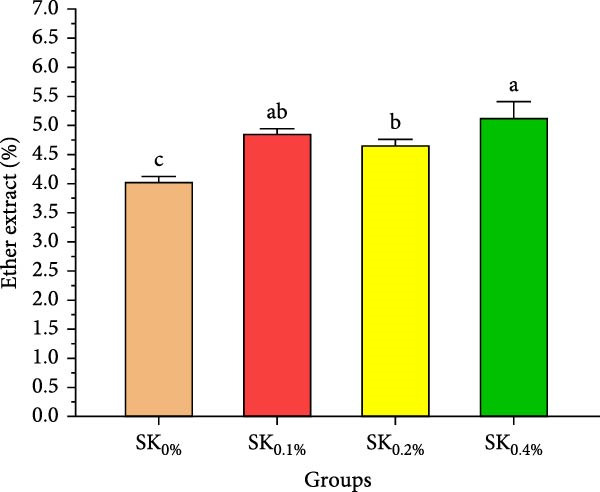
(D)

#### 3.2.3. Digestive Enzyme Activities

Figure 4 reveals the amylase and lipase activities of Pacific whiteleg shrimp fed *S. costatum*. The results showed that, compared to SK_0%_, shrimp‐fed *S. costatum* supplemented groups (SK_0.1%_, SK_0.2%_, and SK_0.4%_) showed significantly (*p* < 0.05) higher amylase activities. Furthermore, in the supplementation groups (SK_0.1%_, SK_0.2%_, and SK_0.4%_), the activities of amylase also showed a nonsignificant increase (*p* < 0.05) with increasing doses of *S. costatum* supplementation (Figure 4A). On the other hand, the SK_0.4%_ group showed a significant (*p* < 0.05) improvement in lipase (Figure 4B) compared to the other groups (SK_0%_, SK_0.1%_, and SK_0.2%_).

Figure 4Digestive enzyme activities of shrimp Pacific whiteleg shrimp‐fed diet supplemented with several levels of *S. costatum*. SK_0%_, SK_0.1%_, SK_0.2%_, and SK_0.4%_: diets supplemented with 0, 1, 2, and 4 g of marine diatom *S. costatum*/kg diet, respectively. The provided data were means ± SD (*n* = 3). Significant differences exist between (*p* < 0.05) letters in the same row. (A) Amylase (U/L) and (B) lipase (U/g).

**Figure:**
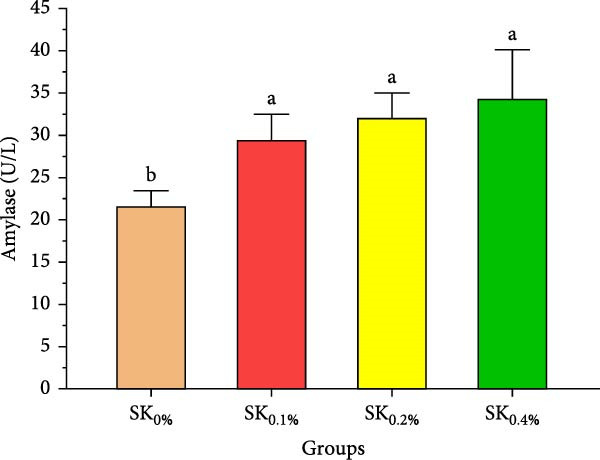
(A)

**Figure:**
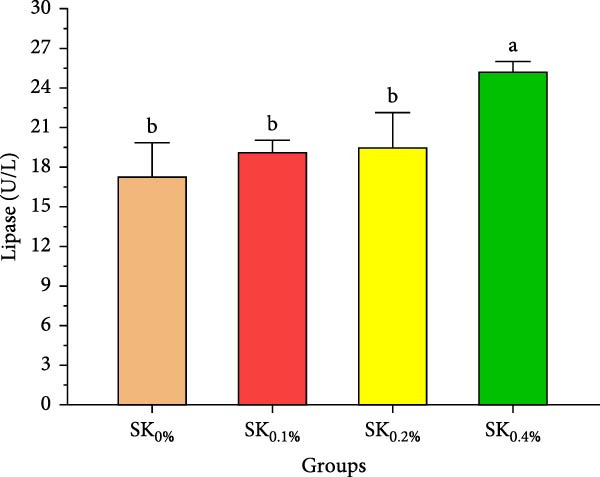
(B)

#### 3.2.4. Antioxidant Activities and Nonspecific Immunity Response

Figure 5 reveals the antioxidant activities of Pacific whiteleg shrimp‐fed diets supplemented by *S. costatum*. Figure 5A,B reveal that, compared to the control group (SK_0%_), shrimp in *S. costatum* supplementation groups (SK_0.1%_, SK_0.2%_, and SK_0.4%_) exhibited the highest significant (*p* < 0.05) CAT and SOD values. While, compared to SK_0%_, *S. costatum* supplementation groups (SK_0.1%_, SK_0.2%_, and SK_0.4%_) exhibited a nonsignificant decrease (*p* < 0.05) in MDA value (Figure 5C). Figure 5D reveals that the SK_0.4%_ group exhibited a significant (*p* < 0.05) improvement in lysozyme value compared to the other groups (SK_0%_, SK_0.1%_, and SK_0.2%_).

Figure 5Antioxidant activities and nonspecific immunity response of *L. vannamei*‐fed diet supported with several levels of *S. costatum*. SK_0%_, SK_0.1%_, SK_0.2%_, and SK_0.4%_: diets supplemented with 0, 1, 2, and 4 g of marine diatom *S. costatum*/kg diet, respectively. The provided data were means ± SD (*n* = 3). Significant differences exist between (*p* < 0.05) letters in the same row. The lack of letters indicates no significance. (A) CAT: Catalase (U/g), (B) SOD: Superoxide dismutase (U/g), (C) MDA: Malondialdehyde (nmol/g), and (D) LZM: Lysozyme (µg/mL).

**Figure:**
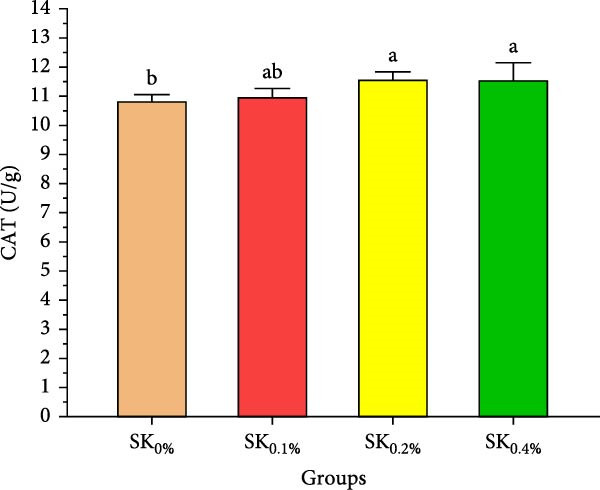
(A)

**Figure:**
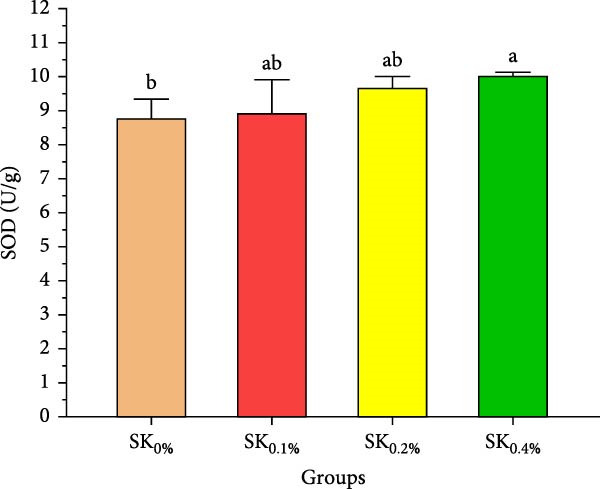
(B)

**Figure:**
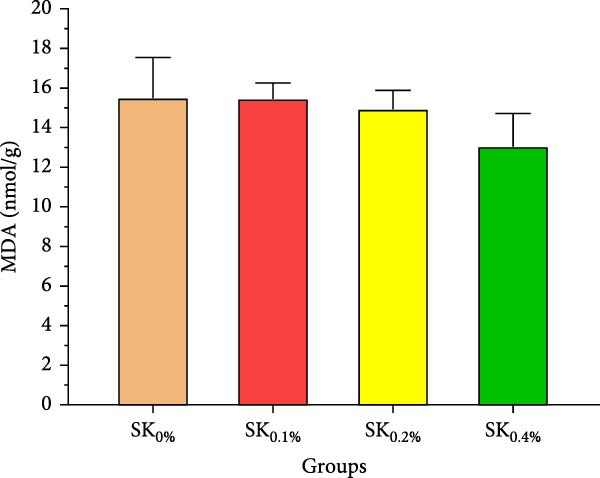
(C)

**Figure:**
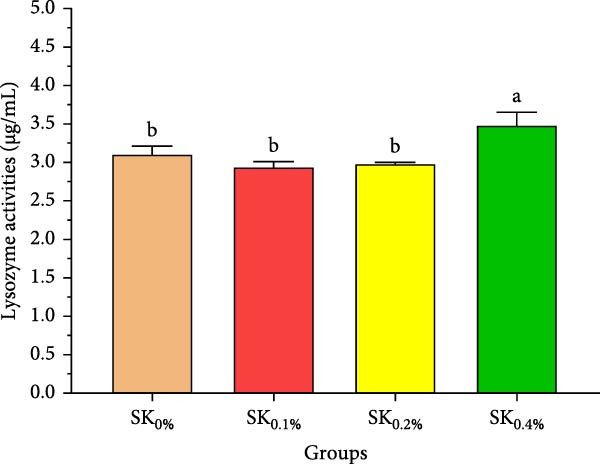
(D)

#### 3.2.5. Immunity‐Related Gene Expressions

Figure 6A–D reveals the relative gene expression levels of immunity‐related genes (A: *P53*, B: *Prx*, C: *PPO1*, and D: *L5H*, respectively) in Pacific whiteleg shrimp fed with dietary supplements containing several levels of *S. costatum*. The results revealed that increasing levels of *S. costatum* led to a significant (*p*  < 0.05) increase in the relative gene expression of the *P53* gene (Figure 6A). SK_0.4%_ revealed the highest significant (*p* < 0.05) increase in the relative gene expression of the *Prx* gene (Figure 6B), compared to other groups (SK_0%_, SK_0.1%_, and SK_0.2%_). As shown in Figure 6C, SK_0.4%_ is the highest significant improvement in the relative gene expression of the *PPO1* gene, followed by SK_0.2%_, and the other groups (SK_0%_ and SK_0.1%_). In contrast, as shown in Figure 6D, there was no significant improvement in the relative gene expression of the *L5H* gene between Pacific whiteleg shrimp‐fed diet supplemented with several levels of *S. costatum* (SK_0.1%_, SK_0.2%_, and SK_0.4%_) and the control group (SK_0%_).

Figure 6Relative gene expression levels of immunity‐related genes in *L. vannamei*‐fed diet supplemented with several levels of *S. costatum*. (A) p53‐like protein isoform delta (*P53*), (B) the peroxiredoxin (*Prx*), (C) prophenoloxidase (*PPO1*), and (D) hemocyanin subunit L5 (*L5H*) genes. SK_0%_, SK_0.1%_, SK_0.2%_, and SK_0.4%_: diets supported with 0, 1, 2, and 4 g of marine diatom *S. costatum*/kg diet, respectively. The provided data were means ± SD (*n* = 3). Significant differences exist between (*p* < 0.05) letters in the same row. The lack of letters indicates no significance.

**Figure:**
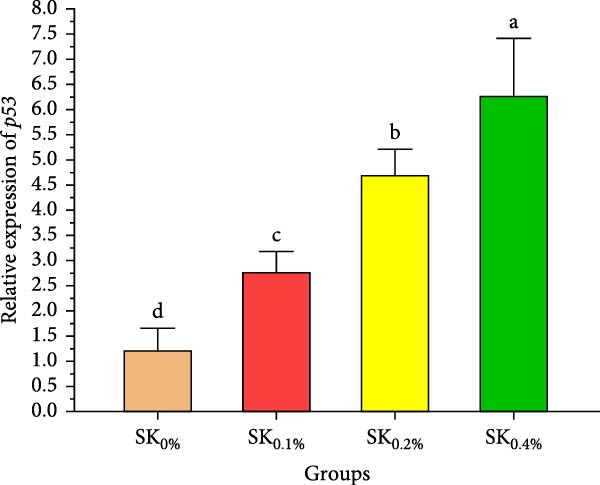
(A)

**Figure:**
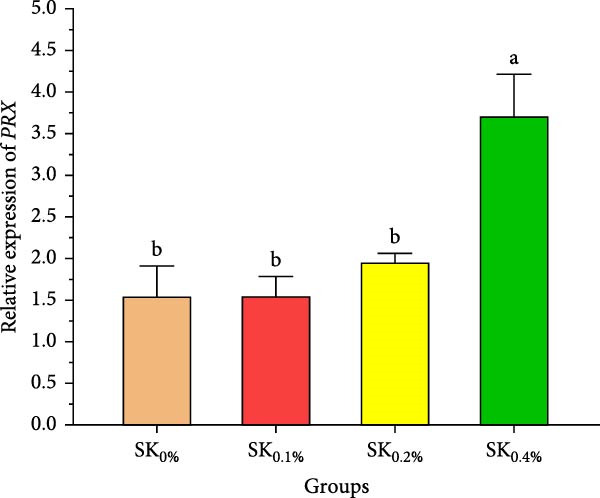
(B)

**Figure:**
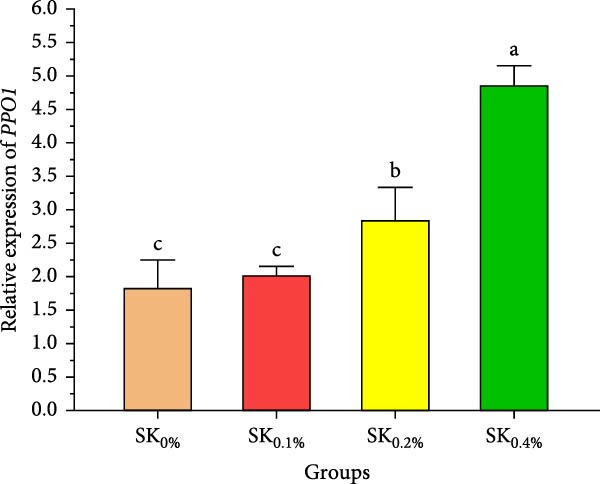
(C)

**Figure:**
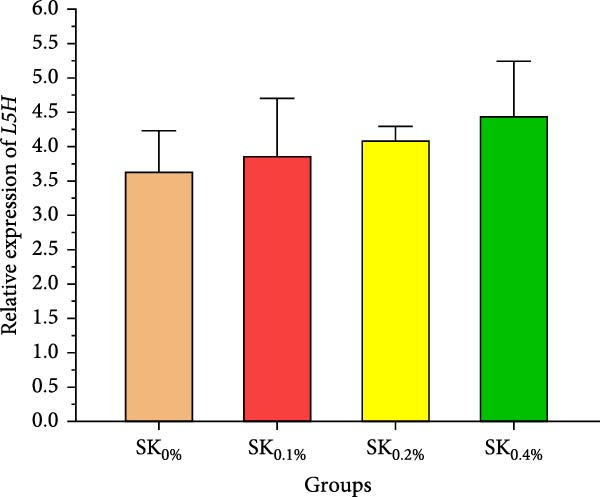
(D)

## 4. Discussion

### 4.1. *S. costatum*

The biochemical compositions of *Skeletonema* species varied based on several conditions, including culture conditions, culture type, culture period, and nutrient limitation [57–59]. The findings of the present study were in accordance with the results by Shang et al. [60] who concluded that, based on culture temperature, the lipid content of *S. dohrnii* varied between 30.97% and 39.23%. In contrast, our findings are in opposition to the findings achieved by Bastos et al. [61], who reported that based on nutrient concentration, the protein and carbohydrate content (%) of *S. costatum* varied between 18.80% and 19.70% and 15.80%–26.20%, respectively.

In the current study, *S. costatum* shows several phytochemical compounds belonging to six phytochemical groups. The study conducted by Javid et al. [62] reported novel applications of myristic acid as an antifungal substrate against *A. niger* and *C. albicans*. As investigated by Liu et al. [63], the myristic acid exhibited strong antioxidant activity in vitro and demonstrated significant hepatoprotective impact against CCl_4_‐induced liver injury *in vivo*. Palmitoleic acid (16:1n−7) is produced from palmitic acid through the action of stearoyl‐CoA desaturase‐1 [55]. This fatty acid has been identified as a lipokine capable of regulating various metabolic processes, including enhancing insulin sensitivity in muscles, promoting *β* cell proliferation, preventing endoplasmic reticulum stress, and stimulating lipogenic activity in white adipocytes [64].

Numerous beneficial impact of palmitoleic acid was observed in cell lines as well as in mouse models [55]. Its exact act in humans remains incompletely understood and is occasionally debated [65]. On the other hand, it is an algicidal bioactive material extensively used against the toxin‐producing dinoflagellate *Alexandrium tamarense*, but its effects on aquatic animals are unclear [66]. Chi et al. [66] study investigated the impacts of PA at concentrations of 20, 40, and 80 mg/L on the immune responses (lysozyme activity, SOD level, MDA level, ACP activity, ROS production, total protein, phagocytic activity) and gene expression of scallop (*Argopecten irradians*). Their study measured various immune parameters at different time points. The study shows that PA exposure at effective concentrations induces varied effects on immune responses and immune‐related gene expression in *A. irradians*, suggesting both beneficial and adverse impacts depending on the timing and concentration of exposure [67].

Marine seagrass (*Thalassodendron ciliatum*) is a rich source of bioactive metabolites, exhibiting notable antioxidant, anti‐inflammatory, and antimicrobial properties, as demonstrated in several studies. GC–MS analysis of this species revealed both saturated and unsaturated fatty acids, with tetradecanoic acid, 12‐methyl‐, methyl ester being particularly abundant (6.07%). This acid is recognized for its potent antibacterial, antifungal, and antiinflammatory activities [53]. 9‐Octadecenoic acid (Z)‐, 2‐ (9‐octadecenyloxy) ethyl ester, (Z)‐ is a fatty acid Octadecenyl ester (FAOE) reported in the extract of passion fruit (*Passiflora edulis*) and showed antibacterial activities against several positive‐ and negative‐gram bacterial strains [48].

Bharathi et al. [47] cited that cholestan‐3‐ol, 2‐methylene‐, (3β, 5α)‐, a saturated tetracyclic steroid that exists in seaweed *Corallina officinalis*, showed strong antiviral activities against SARS‐CoV‐2. Moreover, Losso and Bansode [46] reported that Cholestan‐3‐ol, 2‐methylene‐, (3β, 5α)‐ showed anticancer activities. Chan and Tomlinson [68] showed that trilinolein, a triacylglycerol extracted from Sanchi *Panax pseudoginseng*. This Chinese herb has demonstrated pharmacological properties, including antioxidant properties [50, 68].

Several studies reported that milbemycin is an attractive substrate that enhances the immunity of aquatic animals including Pacific whiteleg shrimp [52] and Nile tilapia (*Oreochromis niloticus*) [69, 70], as well as the plant immunity including strawberry [24] and hot pepper [71]. Zeaxanthin, a potent carotenoid, plays a significant role in enhancing the immune system of aquatic animals [72]. A study by Tan et al. [73] showed that carotenoids play a key role in invertebrate immunity, especially in shrimp immunity. Carotenoid compounds scavenge harmful radicals, reducing immune costs and regulating immune‐related gene expression [74, 75].

### 4.2. Pacific Whiteleg Shrimp

While *S. costatum* is extensively utilized in shrimp hatcheries as live feed for the Pacific whiteleg shrimp and the Giant tiger prawn (*penaeus monodon*) larvae [13, 76], there is no comprehensive study investigating the impact of *S. costatum* as feed additive aquadiet in Pacific whiteleg shrimp nutrition. According to our best knowledge, the study conducted by Lestari et al. [77] is the only study investigating the impact of the dried form of *S. costatum* as a fish meal replacement for Pacific whiteleg shrimp formulated diet. The authors concluded that the inclusion of dried *S. costatum*, as a replacement for fish meal protein, in the Pacific whiteleg shrimp formulated diet at a range of 4.4% to 4.75% exhibited improvement in SR, SGR, FER, and protein efficiency ratio (PER). The current study completely agrees with the results observed by Lestari et al. [77]. Nevertheless, it is widely recognized that many microalgae strains contain significant biologically active components that enhance the growth of Pacific whiteleg shrimp [78] and Nile tilapia [79].

The current study revealed that the percentages of total protein and ether extract have significantly increased with the increase of *S. costatum* supplementation levels. These findings may be attributed to the high protein and lipid levels of marine diatom *S. costatum* (33.50% and 5.95%, respectively). These results agreed with the findings previously observed and concluded that diets supplemented with microalgae levels significantly increase the protein and lipid content of Pacific whiteleg shrimp [80].

The current study showed that shrimp‐fed *S. costatum*‐supplemented groups showed significantly higher amylase activities, compared to the control group. This outcome could be pointed out to the potential of *S. costatum*’s bioactive compounds to boost the activities of digestive enzymes, which would enhance feed digestibility and absorption [81]. Microalgae is a natural part of shrimp feeding regime, and their digestive system is well adapted to digest, absorb, and utilization [82]. Microalgae contains some growth regulators, such as polyamines, and released peptides during digestion could stimulate cholecystokinin release, which in turn enhances the release of digestive enzyme enzymes [83, 84]. On the other hand, dietary *A. platensis* did not significantly affect amylase and lipase activities; however, trypsin and chymotrypsin significantly improved in shrimp‐fed diet with 50% microalgae as fishmeal alternative [85]. Therefore, the relation between dietary microalgae and digestive enzymes release and activity still needs more investigation on the molecular levels to understand their activated synthesis pathway.

Antioxidant activities and nonspecific immunity responses have been investigated as potential biomarkers for assessing the health of aquatic animals [75, 86]. Shrimp primarily rely on nonspecific immunological mechanisms due to their lack of innate immunity [87]. Enzymes like SOD and CAT degrade the cell walls of harmful microbes and scavenge free radicals. MDA is generally utilized as a marker for oxidative stress, signaling an escalation in free radical production [88]. These three hormones play a vital role in both specific and nonspecific immunity in shrimp [72]. Our findings agreed with the findings by Akbari et al. [89], Goda et al. [80], and Zhang et al. [90], indicating that the nonspecific immunity and antioxidant capabilities of Pacific whiteleg shrimp experienced notable enhancements when fed diets incorporating microalgae, *Dunaliella salina*, *Arthrospira platensis*, and *Chlorella vulgaris*, respectively. These improvements can be attributed to the potent content of bioactive compounds of these microalgae, which lead to an improvement in antioxidant activities and nonspecific immune response.

In the same line, the current study reported that marine diatom *S. costatum* contains a significant amount of several bioactive materials that show antioxidant activities, such as zeaxanthin, a rich antioxidant compound that is well‐known for its ability to improve the shrimp’s immune response, particularly by influencing key biochemical markers such as CAT, SOD, lysozyme, and MDA [91]. Mabrouk et al. [92] evaluated the effects of the inclusion of the benthic freshwater diatom *Amphora coffeaeformis* into the dietary supplementation of tilapia broodstock, examining blood chemistry, steroid hormone concentration, and seed production health. The authors concluded that *A. coffeaeformis* supplementation levels of 4% to 6% led to significant improvements in the studied indicators of Nile tilapia broodstock, suggesting the potential efficacy of the benthic freshwater diatom *A. coffeaeformis* as a beneficial aquafeed additive during the spawning season. The study by Sandeep et al. [93] assessed the impact of the nutritional value and antimicrobial potential of the marine diatom *T. weissflogii* and the marine green microalgae *Tetraselmis* sp. concentrates on Pacific whiteleg shrimp PL. *Tetraselmis* sp. displayed superior inhibition against *Vibrio parahaemolyticus*. They concluded that the feeding trials revealed enhanced growth and immunity with *Tetraselmis* diet inclusion, indicating its promise for early shrimp development [93].

Peroxiredoxin (*Prx*) is an antioxidant gene that plays a crucial key in protecting cells from oxidative stress by reducing hydrogen peroxide and organic peroxides. In shrimp, *Prx* is involved in maintaining cellular homeostasis during immune responses to pathogenic bacteria and toxic environmental stressors such as giant tiger r shrimp [94] and Pacific whiteleg shrimp [95]. Prophenoloxidase (*PPO1*) is a critical gene in the melanization cascade, which is an immune defense mechanism in innate immunity in giant tiger prawn [96] and Pacific whiteleg shrimp [52]. *ProPO* plays a vital role in the shrimp’s defense system [97]. Therefore, *PPO1* is essential for both the immune defense and tissue repair processes in shrimp [98]. The p53‐like protein isoform delta (*p53*) is part of the broader *p53* family, which is well‐known for its tumor‐suppressive properties [99]. In shrimp, the *p53* protein is involved in regulating the cell cycle and apoptosis, particularly in response to stressors such as pathogen infection or cellular damage [100]. The shrimp’s immune system relies on this function of *p53* to eliminate infected or damaged cells, preventing the spread of pathogens [101]. The significant upregulation of these immune genes could be attributed to the role of *S. costatum*’s bioactive compounds (e.g., zeaxanthin, palmitoleic acid) on antioxidant status, which subsequently enhances immune responses in Pacific whiteleg shrimp [52]. These findings were confirmed by significant increase in *Prx*, *p53*, and *PPO1* expression alongside elevated antioxidant enzyme activities (e.g., SOD, CAT) in shrimp fed *S. costatum*, which in turn reduces cellular stress and induces immune gene activation. However, this pathway still merits molecular investigation.

Hemocyanin subunit (*L5H*) is a copper‐based respiratory protein found in the hemolymph (blood) of many arthropods, including shrimp. The hemocyanin subunit L5 (*L5H*) plays an important role in both oxygen transport and immune defense in Pacific whiteleg shrimp [102]. In response to immune challenges, the expression of *L5H* increases, indicating its involvement in the immune response [103]. Increases in the expression of *L5H* also help in the recognition of pathogens and are thought to play a role in the activation of the shrimp’s immune system [104]. As a part of the innate immune defense, *L5H* aids in the clearance of pathogens from the hemolymph, thereby preventing systemic infections. Its ability to bind to pathogens and enhance the immune response makes *L5H* a critical protein in the shrimp’s defense mechanisms [52].

The results found that the increasing levels of *S. costatum* led to a significant increase in the relative gene expression of the *P53*, *Prx*, and *PPO1* genes. The current findings agreed with several previous studies indicating that incorporating algal cells (microalgae and/or seaweeds) or their derivatives into the shrimp diet has improved the regulation of immune‐related genes in several aquatic animals [52, 105, 106].

## 5. Conclusion

This study demonstrates the beneficial effects of dietary inclusion of the marine diatom species *S. costatum* on the growth, antioxidant activities, immunological responses, digestive enzymes, and immunity‐associated gene expressions of Pacific whiteleg shrimp PL. The results clearly indicate that higher dietary concentrations of *S. costatum* (particularly at 0.4% inclusion) significantly enhance growth indicators, immunological responses, digestive enzyme activities, and antioxidant activities, compared to the control group. Shrimp feed at 0.4% exhibited the highest improvements in key physiological parameters such as amylase, CAT, SOD, lipase, lysozyme activities, and immune‐related gene expressions (*p53*, *Prx*, and *PPO1*). These findings suggest that *S. costatum* can serve as a promising, sustainable aquafeed additive to boost shrimp health, immunity, and growth. Further studies are needed to investigate the long‐term impact and optimal dietary concentrations of *S. costatum* for different shrimp species.

## Conflicts of Interest

The authors declare no conflicts of interest.

## Author Contributions

**Mohamed Ashour:** conceptualization, formal analysis, validation, investigation, software, resources, methodology, writing – review and editing, writing – original draft preparation, project administration. **Einar Ringø:** visualization, resources, supervision, and writing – review and editing. **Mohamed M. Mabrouk:** conceptualization, methodology, formal analysis, software, investigation, resources, and writing – original draft preparation. **Ahmed I. A. Mansour:** conceptualization, methodology, formal analysis, resources, software, writing – original draft preparation, validation, investigation. **Mohammed A. E. Naiel:** methodology. **Abdallah Tageldein Mansour:** visualization, funding acquisition, supervision, writing – review and editing. **Rabee M. A. Gheetas:** software, investigation, resources, methodology, writing – original draft preparation. **Ehab Mohamed:** visualization, resources, supervision, writing – review and editing. **Ahmed F. Abdelhamid:** conceptualization, methodology, software, investigation, resources, validation, and writing – original draft preparation. All authors have read and agreed to the published version of the manuscript.

## Funding

The authors express their gratitude to the support of the Deanship of Scientific Research, Vice Presidency for Graduate Studies and Scientific Research, King Faisal University, Saudi Arabia (KFU251915).

## Data Availability

The data that support the findings of this study are available from the corresponding author upon reasonable request.
